# Ophthalmology and Otology

**Published:** 1897-04

**Authors:** 


### SELECTIONS AND ABSTRACTS.

### OPHTHALMOLOGY AND OTOLOGY.

Edited by Alex. W. Stirling, M.D.

### Causes of Glaucoma.

Dr. Leartus Connor, of Detroit, discusses this subject in *Journal of the American Medical Association* (November 14, 1896), and closes his paper with the following conclusions:

1. Glaucoma is a disease rather than a deformity, like hernia or astigmatism.

2. The disease glaucoma has two distinct factors: one including changes in the connective tissues of the body, including the outflow spaces of the posterior chamber of the eye; the other so altering the secretion of the posterior chamber that it clogs the crippled outflow connective tissue spaces.

3. The first factor results from the long continued action of impure blood upon the connective tissue elements of the outflow spaces, the second from a variety of conditions acting through the nervous vascular, digestive and muscular systems, or through local strain of the eye, as in presbyopia, uncorrected astigmatism, or intraocular tumors, dislocated lens, or from the use of mydriatics.

4. Impure blood may result from many distinct diseases, as gout, rheumatic gout, syphilis, or from chronic overloading of the body with food in excess of its assimilative powers.

5. In simple glaucoma the connective tissue at the optic papilla is so attacked as to induce an excavation of the optic disc. If the anterior outflow space remains patent, there may be no symptoms except diminishing field of vision and the excavation, but if the anterior outflow becomes obstructed, increase of tension and other glaucomatous symptoms appear.

6. In acute inflammatory glaucoma the anterior outflow spaces are suddenly closed, including all the typical symptoms of a glaucomatous outburst.

7. In subacute glaucoma the obstruction to the anterior outflow is less sudden and complete, and hence the symptoms are less severe and startling.

8. In chronic inflammatory glaucoma the obstruction is more complete and permanent, and so the effects are more hopelessly destructive—glaucoma absolute marking the final stage when vision is totally lost.

9. In hemorrhagic glaucoma the outburst is complicated by rupture of weakened intraocular blood-vessels, so that hope of relief is slight.

10. In secondary glaucoma the same obstruction to the outflow channels occurs. This may be due to an intraocular tumor, a dislocated lens, a lens swollen after discission, or to occlusion of the pupil, etc.

---

### SCOPOLAMINE IN PLASTIC IRITIS.

Dr. Charles A. Oliver thus concludes a paper on this subject (*American Journal Medical Sciences,* November):

1. Hydrobromate of scopolamine is of the greatest value in the local treatment of the various forms of plastic iritis.

2. The primary reparative action and quieting power of hydrobromate of scopolamine as compared with those of similar doses of sulphate of atropine in the treatment of plastic iritis are much more promptly obtained with the former than the latter drug, this being true in the majority of cases, even when the latter drug is used in dosages that are equal to quadruple or quintuple the strengths that are employed of the former drug.

3. The duration of the healing and soothing powers of hydrobromate of scopolamine as compared with those of equal doses of sulphate of atropine in cases of plastic iritis does not seem to be so lasting with the former as with the latter drug, this being true in the majority of cases even when the latter drug is used in four or five times the strength-doses as the former.

4. For quick and active measures, which are so eminently necessary in incipient cases of plastic iritis, and during the early stages of inflammatory reaction, hydrobromate of scopolamine is to be preferred to sulphate of atropine.

5. Where prolonged use of such drug is necessary, as in many cases of the chronic form of the disease with subacute exacerbations, the alternate employment of hydrobromate of scopolamine and sulphate of atropine seems empirically to be the best method of local administration that has been devised.

6. As clinically employed, the best salt of the alkaloid seems to be the hydrobromate; the best method of instillation appears to be that which is accomplished by dropping the solution upon the upper corneal limbus whilst the lower punctum is everted and the corresponding canaliculus is pressed upon; and the most efficient amount to be used at one sitting is two drops of a one-tenth of 1 per cent. strength (1 to 500), repeated, if necessary, as often as three times during the course of an hour, and preceded, when desired, as in some instances where there are much irritation and pain, by two drops of a 2 per cent. solution of hydrochlorate of cocaine a few minutes before each instillation of the scopolamine.

---

### FOREIGN BODIES IN THE EAR.

Preotraschensky (Glasgow *Med. Jour.*, *Laryngoscope*), after calling attention to the unpleasant and at times serious complications which follow unskillful attempts at removal of foreign substances from the external auditory canal, offers the following suggestions:

1. An unskilled person should never attempt the instrumental extraction of a foreign body.

2. Foreign bodies reach the middle ear almost solely as the result of clumsy attempts at removal.

3. The foreign body usually does less harm to the ear than its extraction by an unpracticed hand.

4. The changes produced by the presence of a foreign body in the ear cannot be estimated by the length of time during which it has remained there.

5. The injection of warm water is an infallible means of securing the evacuation of any foreign body from the ear; irrigation with alcohol may be further necessary to prevent swelling of the intruder.

6. There is no indication to expedite the removal of foreign bodies which are giving rise to no troublesome symptoms.

7. In inflammatory processes caused by necrosis from unskilled attempts at extraction, expectant treatment suffices as long as no dangerous symptoms are present. Turpentine and ether for larvæ; these oils cause inflammatory conditions, so we had better resort to instrumental means.

When the foreign element proves to be of the vegetable kingdom, water would better be omitted from the methods suggested, as such a substance readily absorbs moisture, and so increases in size, making its extraction even more difficult than before.—*Med. Standard.*

---

### PREVENTION OF OPHTHALMIA NEONATORUM.

Zimmerman (*Medicine*) summarizes the prophylaxis of ophthalmia neonatorum as follows:

1. Men affected with gonorrhea or gleet ought not to be allowed to marry.

2. The vagina of a woman suspected of gonorrhea should be cleansed with antiseptic solutions before and during labor.

3. As soon as the child is delivered, the lids and surrounding parts should be dried with aseptic cotton or gauze and then washed with boiled water or sublimate solution, 1 to 5,000—never with sponges or with the water in which the child has been bathed.

After the bath the eyes are treated according to Crede's method: The lids are held apart with two clean fingers, and a drop of a two-per-cent. solution of nitrate of silver is dropped on the cornea from a glass rod. Then the eyes are left alone; especially in the next twenty-four to thirty-six hours, if a slight redness or swelling of the lids with mucous discharge should occur, the instillation is not to be repeated.

4. The most careful cleanliness in washing and handling the child will prevent secondary infection.

5. If, however, the eyes show the least sign of pus on the subsequent days, it is the duty of the midwife or nurse to send at once for a physician who is familiar with the disease.

6. Any nurse or midwife who, from ignorance or obstinacy, fails to report such a case at once should be severely fined and excluded from further practice.

If these rules are strictly adhered to, says Zimmerman, ophthalmia neonatorum will be prevented and many eyes saved from blindness.—*Medical Standard.*

### OPERATION FOR MASTOIDITIS, COMPLICATED BY HEMORRHAGE FROM THE LATERAL SINUS: RECOVERY.

The patient, a boy, aged 12 years, was brought under the care of the author suffering from pain around the ear, an outstanding auricle and discharge. Upon examination a perforation was found in the posterior superior quadrant of the membrana tympani, redness and edema of the mastoid region, auricle prominent, temperature 100° F. Under treatment the symptoms improved for a few days, after which a relapse took place. Under chloroform the usual incision behind the auricle was made. Upon exposing the bone a small fistula was found above the superior wall of the external meatus and about three-quarters of an inch behind it. Before any exploration could be made there was a sudden rush of dark colored blood, which was recognized as coming from the lateral sinus. A firm compress of iodoform gauze was at once put on and the patient carefully watched. Upon the tenth day the bandage was removed, and the operation of opening the antrum was performed. In the author's opinion the cause of the hemorrhage was that there had been an erosion of the walls of the lateral sinus, as the pus found its way through the bone, and that when the periosteum was removed the support of the weakened vessel thereby was taken away, and the blood forced its way through the eroded walls.—C. R. DUFOUR, in *Laryngoscope*.—*Med. Chronicle.*

---

### HEADACHES FROM EYE STRAIN.

Dr. S. Weir Mitchell, in an article on the subject (*Medical News*) formulates the following conclusions: (1) There are many headaches which are due directly to disorders of the refractive or accommodative apparatus of the eyes. (2) In some instances the brain symptom is often the most prominent and sometimes the sole prominent symptom of the eye trouble, so that while there may be no pain or sense of fatigue in the eye, the strain with which it is used may be interpreted solely by occipital or frontal headache. (3) The long continuance of eye troubles may be the unsuspected source of insomnia, vertigo, nausea and general failure of health. (4) In many cases the eye trouble becomes suddenly mischievous, owing to some failure of the general health, or to increased sensitiveness of the brain from moral or mental causes.—*Medical Times.*

